# Prevalence and Predictors of Adolescent Tobacco Use in the Russian Federation: Insights From a Nationwide Survey

**DOI:** 10.1002/puh2.70254

**Published:** 2026-04-27

**Authors:** Ashfaq Ahmad Shah Bukhari, Syed Sikandar Shah, Mouhammed Sleiay, Mahmoud M. Ali

**Affiliations:** ^1^ Department of Physiology RAK College of Medical Sciences RAK Medical and Health Sciences University Ras Al Khaimah UAE; ^2^ Department of Clinical Pharmacy and Pharmacology Rak College of Pharmacy RAK Medical and Health Sciences University Ras Al Khaimah UAE; ^3^ Faculty of Medicine Hama University Hama Syria; ^4^ Nabaruh Health Administration Directorate of Health Affairs Nabaruh, Dakahlia Governorate Egypt; ^5^ Faculty of Pharmacy Al‐Azhar University Assiut Egypt

**Keywords:** adolescent tobacco use, Global Youth Tobacco Survey (GYTS), public health, Russia, tobacco use, tobacco use prevalence

## Abstract

**Background:**

Tobacco use is a leading cause of preventable disease and death worldwide. In Russia, despite tobacco‐control measures, adolescent tobacco use remains a significant public health concern.

**Objective:**

This study aimed to assess the prevalence and predictors of tobacco use among adolescents aged 13–15 years in the Russian Federation using data from the Global Youth Tobacco Survey (GYTS).

**Methods:**

The present study was a cross‐sectional study based on secondary analysis of data from the 2021 Russian Federation GYTS. Descriptive statistics, bivariate analysis, and multivariable logistic regression were used to evaluate sociodemographic and attitudinal factors associated with adolescent tobacco use.

**Results:**

The findings showed that the prevalence of adolescent tobacco use was 16.3% among 13‐year‐olds, to 21.4% among 14‐year‐olds, and 29.6% among 15‐year‐olds (*p* < 0.001). Male adolescents had a higher prevalence of tobacco use than females (22.7% vs. 20.8%, *p* = 0.009). Tobacco use prevalence also increased by school level, reaching 29.0% among ninth‐grade students, compared with 17.2% and 20.7% among seventh‐ and eighth‐grade students, respectively (*p* < 0.001). Weekly spending was strongly associated with tobacco use, with prevalence rising to 36.0% among adolescents spending 1000 rubles or more per week. In multivariable analysis, adolescents spending 1000 rubles or more per week had over twice the odds of tobacco use (adjusted odds ratio [OR]: 2.03; 95% confidence interval [CI]: 1.75–2.46; *p* < 0.001). Positive tobacco use expectancies were also strongly associated with tobacco use, with adjusted ORs reaching 10.79 among adolescents who agreed that they might enjoy tobacco use.

**Conclusions:**

The findings of this study highlight that adolescent tobacco use in Russia continues to be a significant public health issue, characterized by an increasing prevalence with age, an emergence of a relatively small sex difference in prevalence, and strong associations with socioeconomic factors and positive tobacco use expectancies.

## Graphical abstract

Adolescent tobacco use in Russia increases with age and is strongly associated with higher weekly spending and positive smoking attitudes, emphasizing the need for strengthened tobacco‐control policies and youth prevention strategies.

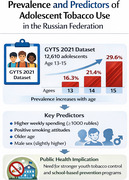

## 1 Introduction

Tobacco use remains one of the most pervasive and preventable causes of disease and premature death worldwide [1]. Tobacco use prevalence has declined globally and in several WHO regions following the implementation of comprehensive tobacco‐control policies, including in the WHO European Region and the Region of the Americas [2, 3, 4]. However, the Russian Federation, as part of the WHO European Region, continues to face a substantial tobacco burden [2, 3, 4].

Adolescent tobacco use remains a significant public health concern in the Russian Federation [5]. Data from the Global Youth Tobacco Survey (GYTS) indicate a notable decline in tobacco consumption among adolescents in the Russian Federation, decreasing from 27.3% in 2004 to 11.0% in 2021 [6]. This reduction is attributed to comprehensive tobacco‐control measures, including the 2013 Federal Law aimed at protecting citizens from tobacco smoke and its consequences [7]. These measures included bans on tobacco use in public places, restrictions on tobacco advertising, promotion, and sponsorship, and increased taxes on tobacco products, which may also have contributed to reductions in youth tobacco use [8].

Understanding the predictions of adolescent tobacco use is crucial for developing effective prevention strategies. Studies conducted in various countries have identified several factors associated with tobacco use among adolescents aged 13–15 years. For instance, research in the United States and Russia found that peer influence and sensation‐seeking behavior were important predictors of adolescent tobacco use [9]. Furthermore, tobacco use behavior may also be influenced by parents’, teachers’, and friends’ tobacco use, as reported in a study in Slovakia [10]. Furthermore, tobacco use behaviors might be attributed to parents’, teachers’, and friends’ tobacco use as reported in a study in Slovakia [11].

Our study aimed to assess the prevalence and predictors of tobacco use among adolescents aged 13–15 years in the Russian Federation. By analyzing GYTS data, we sought to inform policy and intervention efforts to reduce tobacco use in this population.

## 2 Materials and Methods

This study was a cross‐sectional secondary analysis of data from the 2021 Russian Federation GYTS, a standardized, school‐based survey designed to monitor tobacco‐related behaviors among adolescents. The present study used the existing GYTS dataset rather than collecting primary data.

### 2.1 Sampling

The 2021 GYTS used a standardized two‐stage cluster sampling design. In the first stage, schools were selected with probability proportional to enrollment size. In the second stage, classes within selected schools were randomly selected, and all students in those classes were eligible to participate [12]. In the Russian Federation, sampling and weighting were stratified by gender, and regional data were merged into a national dataset. The broader surveyed sample included 13,497 eligible students from grades 7–9. For the present secondary analysis, we restricted the analytic sample to 12,610 adolescents aged 13–15 years, in accordance with the study objective [12].

### 2.2 Study Variables

The primary outcome variable was current tobacco use. Explanatory variables included sociodemographic characteristics and tobacco use‐related attitudinal factors available in the GYTS dataset. Sociodemographic variables included age, sex, school level, and weekly personal spending money. Tobacco use‐related attitudinal variables included responses to the statements “I think I might enjoy smoking a cigarette” and “Do you think smoking tobacco helps people feel more comfortable or less comfortable at celebrations, parties, or in other social gatherings?” These variables were selected because they were available in the dataset and were relevant to the study objective of examining the prevalence and predictors of adolescent tobacco use.

### 2.3 Questionnaire

GYTS uses a standard core questionnaire, supplemented by optional country‐specific items, to measure key tobacco‐control indicators. The dataset includes information on tobacco use, cessation, secondhand smoke exposure, tobacco advertising and promotion, product access, and tobacco‐related knowledge and attitudes. In the original survey, data were collected using anonymous self‐administered questionnaires completed on scannable response sheets [13].

### 2.4 Statistical Analysis

Data from the 2021 GYTS secondary dataset were analyzed using SPSS version 27. Descriptive statistics were used to summarize the characteristics of the study sample as frequencies and percentages. Bivariate analyses using chi‐square tests assessed associations between explanatory variables and tobacco use status. Unadjusted and adjusted odds ratios (ORs) with 95% confidence intervals (CIs) were estimated using logistic regression to identify predictors of tobacco use. Multivariable models were adjusted for age, sex, and school level. Statistical significance was set at *p* < 0.05. Model fit was assessed using the Hosmer–Lemeshow goodness‐of‐fit test, and missing data were handled by pairwise exclusion.

## 3 Results

The study examined the prevalence of tobacco use and the sociodemographic and attitudinal predictors of tobacco use among adolescents aged 13–15 years in the Russian Federation. A total of 13,497 eligible students from intermediate level (7, 8, and 9) completed the survey, of which 12,610 were aged 13–15 years.

The findings showed that the prevalence of adolescent tobacco use was 16.3%, 21.4%, and 29.6% among adolescents aged 13, 14, and 15 years, respectively (*p* < 0.001). Males had a higher prevalence of tobacco use than females (22.7% vs. 20.8%, *p* = 0.009). Tobacco use prevalence also increased by school level, from 17.2% in seventh grade to 29.0% in ninth grade (*p* < 0.001). Weekly personal spending money was significantly associated with tobacco use prevalence, increasing from 16.2% among adolescents spending less than 250 rubles to 36.0% among those spending 1000 rubles or more (*p* < 0.001) (Table 1).

**TABLE 1:** Current prevalence of adolescents’ tobacco use by their sociodemographic characteristics.

Characteristics	Tobacco users No.	Percentages^a^	Non‐users No.	Percentages^a^	*p* value
**Age group in years**	2645		9497		<0.001^**^
13	699	16.3	3598	83.7	
14	989	21.4	3623	78.6	
15	957	29.6	2276	70.4	
**Sex**	2622		9447		0.009^**^
Male	1351	22.7	4595	77.3	
Female	1271	20.8	4852	79.2	
**School level**	2623		9423		<0.001^**^
7 classes	745	17.2	3587	82.8	
8 classes	897	20.7	3437	79.3	
9 classes	981	29.0	2399	71.0	
**Spend on yourself on an average week**	2633		9464		<0.001^**^
I usually do not have any spending money	391	19.9	1570	80.1	
Less than 250 rub	564	16.2	2901	83.8	
250–399 rub	506	19.4	2098	80.6	
400–599 rub	422	24.3	1314	75.7	
600–799 rub	199	26.8	543	73.2	
800–999 rub	169	32.3	354	67.7	
1000 rub or more	384	36.0	684	64.0	

^a^ Percentages are rounded.

** Significant.

Analysis of the association between sociodemographic factors and tobacco use showed that older adolescents had significantly higher odds of tobacco use. Compared with 13‐year‐olds, the odds of tobacco use were higher among 14‐year‐olds (OR = 1.405, 95% CI: 1.262–1.564, *p *< 0.001) and 15‐year‐olds (OR = 2.164, 95% CI: 1.937–2.418, *p *< 0.001). Male adolescents were also more likely to smoke compared with females (OR = 1.122, 95% CI: 1.029–1.224, *p* = 0.009). Higher school grades were associated with increased odds of tobacco use, particularly among ninth‐grade students (OR = 1.969, 95% CI: 1.767–2.194, *p *< 0.001). In addition, adolescents with higher weekly spending money showed progressively higher odds of tobacco use, reaching more than twice the risk among those spending 1000 rubles or more (OR = 2.254, 95% CI: 1.908–2.664, *p *< 0.001) (Table 2).

**TABLE 2:** Association of sociodemographic factors with the risk of tobacco use.

Characteristics	Tobacco users	Non‐users	OR	95% CI for OR	*p* value
**Age group in years**	2645	9497			
13	699	3598	1	Ref.	
14	989	3623	1.405	1.262–1.564	<0.001
15	957	2276	2.164	1.937–2.418	<0.001
**Sex**	2622	9447			
Male	1351	4595	1.122	1.029–1.224	0.009
Female	1271	4852	1	Ref.	
**School level**	2623	9423			
7 classes	745	3587	1	Ref.	
8 classes	897	3437	1.257	1.128–1.400	<0.001
9 classes	981	2399	1.969	1.767–2.194	<0.001
**Spend on yourself on an average week**	2633	9464			
I usually do not have any spending money	391	1570	1	Ref.	
Less than 250 rub	564	2901	0.778	0.674–0.897	<0.001
250–399 rub	506	2098	0.968	0.836–1.122	0.669
400–599 rub	422	1314	1.290	1.103–1.507	<0.001
600–799 rub	199	543	1.472	1.209–1.791	<0.001
800–999 rub	169	354	1.917	1.547–2.375	<0.001
1000 rub or more	384	684	2.254	1.908–2.664	<0.001

Abbreviations: CI, confidence interval; OR, odds ratio.

Smoking‐related perceptions and social attitudes were strongly associated with cigarette smoking among adolescents. Adolescents who reported that they currently smoke cigarettes had markedly higher odds of agreeing that they might enjoy smoking (OR = 7.735, 95% CI: 6.576–9.099, *p *< 0.001). Those who agreed or strongly agreed with the statement “I think I might enjoy smoking a cigarette” had significantly higher odds of smoking compared with those who strongly disagreed. Additionally, adolescents who believed that smoking makes people feel more comfortable at celebrations or social gatherings had higher odds of smoking (OR = 1.757, 95% CI: 1.587–1.946, *p *< 0.001), whereas those who believed it makes people feel less comfortable had lower odds (OR = 0.846, 95% CI: 0.756–0.946, *p* = 0.003) (Table 3).

**TABLE 3:** Association of tobacco use‐related factors with the risk of adolescents’ tobacco use.

	Tobacco users	Non‐users	OR	95% CI	*p* value
**Do you agree or disagree with the following: “I think I might enjoy smoking a cigarette”**	2620	9354			
I currently smoke cigarettes	384	351	7.735	6.576–9.099	<0.001
Strongly agree	188	178	7.468	6.006–9.285	<0.001
Agree	300	197	10.767	8.866–13.076	<0.001
Disagree	940	2915	2.280	2.055–2.530	<0.001
Strongly disagree	808	5713	1	Ref.	
**Do you think smoking tobacco helps people feel more comfortable or less comfortable at celebrations, parties, or in other social gatherings?**	2575	9233			
More comfortable	990	2304	1.757	1.587–1.946	<0.001
Less comfortable	598	2892	0.846	0.756–0.946	0.003
No difference	987	4037	1	Ref.	

Abbreviations: CI, confidence interval; OR, odds ratio.

Multivariable analysis identified several significant predictors of adolescent cigarette smoking after adjustment for age, sex, and school level. Agreement with the statement “I think I might enjoy smoking a cigarette” remained a strong predictor, particularly among those who agreed (OR = 10.785, 95% CI: 8.854–13.138, *p *< 0.001) or strongly agreed (OR = 7.521, 95% CI: 6.015–9.404, *p *< 0.001), compared with those who strongly disagreed. However, the difference between these two categories should be interpreted cautiously, as both were strongly associated with smoking, and their CIs overlapped. Perceiving smoking as making people more comfortable in social gatherings was also associated with higher odds of smoking (OR = 1.718, 95% CI: 1.549–1.905, *p *< 0.001). Furthermore, higher levels of weekly spending money independently predicted smoking, with adolescents spending 1000 rubles or more showing approximately double the odds of smoking compared with those without spending money (OR = 2.027, 95% CI: 1.747–2.458, *p *< 0.001) (Table 4).

**TABLE 4:** Predictors of adolescents’ tobacco use.

	Tobacco users	Non‐users	OR^a^	95% CI	*p* value
**Do you agree or disagree with the following: “I think I might enjoy smoking a cigarette”**	2620	9354			
I currently smoke cigarettes	384	351	7.848	6.653–9.257	<0.001
I strongly agree	188	178	7.521	6.015–9.404	<0.001
Agree	300	197	10.785	8.854–13.138	<0.001
Disagree	940	2915	2.253	2.027–2.505	<0.001
Strongly disagree	808	5713	1	Ref.	
**Do you think smoking tobacco helps people feel more comfortable or less comfortable at celebrations, parties, or in other social gatherings?**	2575	9233			
More comfortable	990	2304	1.718	1.549–1.905	<0.001
Less comfortable	598	2892	0.872	0.778–0.978	0.019
No difference	987	4037	1	Ref.	
**Spend on yourself on an average week**	2633	9464			
I usually do not have any spending money	391	1570	1	Ref.	
Less than 250 rub	564	2901	0.787	0.681–0.910	0.001
250–399 rub	506	2098	0.944	0.813–1.097	0.453
400–599 rub	422	1314	1.224	1.044–1.435	0.013
600–799 rub	199	543	1.410	1.154–1.721	<0.001
800–999 rub	169	354	1.814	1.459–2.256	<0.001
1000 rub or more	384	684	2.027	1.747–2.458	<0.001

Abbreviations: CI, confidence interval; OR, odds ratio.

^a^ OR adjusted by age, sex, school level.

## 4 Discussion

In this study, we examined the prevalence and predictors of tobacco use among Russian adolescents aged 13–15 years, using data from the 2021 GYTS. Our key findings include (1) an overall current tobacco use prevalence of 12.1% (16.3% at age 13; 21.4% at age 14; 29.6% at age 15); (2) a clear sociodemographic gradient higher odds of tobacco use among older adolescents, males, ninth‐grade students, and those with greater weekly spending; and (3) very strong associations between positive tobacco use expectancies (enjoyment, social comfort) and actual tobacco use behavior (adjusted ORs up to ∼10).

Our results demonstrate a steep age gradient: 13‐year‐olds had a 16.3% tobacco use prevalence, rising to 29.6% by age 15 (*p* < 0.001). This pattern mirrors findings from Ma et al., who reported that tobacco use prevalence among 13–15‐year‐olds increased substantially with age, with adjusted odds of current tobacco use nearly doubling between ages 13 and 15 [14]. In Russia specifically, Ross found that each additional year of age was associated with a significant increase in the odds of tobacco use initiation among Moscow students [15].

Adolescents aged 13–15 years in our study demonstrated a marked age gradient in tobacco use prevalence, increasing from 16.3% at age 13 to 29.6% at age 15. This pattern may be more specifically explained by age‐related increases in autonomy, reduced parental monitoring, and easier access to cigarettes through peers and retail sources among older adolescents. Older adolescents are also more likely to spend unsupervised time in social environments where exposure to tobacco use and opportunities for experimentation are greater. Accordingly, the higher tobacco use prevalence observed among older adolescents is likely related to increasing access and exposure opportunities with age [13, 16, 17, 18].

Male adolescents in our sample had a higher tobacco use prevalence than females (22.7% vs. 20.8%; *p* = 0.009). Previous studies have reported somewhat higher smoking rates among male adolescents, often attributed to social, behavioral, and cultural influences [19, 20, 21, 22, 23]. However, the difference observed in our study was relatively small, suggesting that tobacco use prevention efforts should target both boys and girls.

Our finding of a significant relationship between weekly spending and tobacco use (adjusted OR 2.03 for ≥1000 rubles vs. none; *p* < 0.001) is consistent with international literature showing that pocket money predicts adolescent tobacco use.

A study has shown that higher disposable income or weekly spending money among adolescents lowers financial barriers to tobacco experimentation and use. First, adolescents with more spending money can more readily afford cigarettes or single cigarettes, directly increasing purchase opportunities and trial rates [24]. Second, higher personal income has been associated with tobacco use even after controlling for family socioeconomic status, suggesting that the ability to spend on cigarettes may facilitate tobacco use uptake [24]. In addition, greater spending money may reflect increased autonomy and more unsupervised social time, contexts in which adolescents are more likely to encounter tobacco use opportunities through peers. Collectively, these mechanisms may help explain why weekly spending is associated with both tobacco use experimentation and regular tobacco use among adolescents.

One of the notable findings in our study was the strong association between positive tobacco use expectancies and adolescent tobacco use. Adolescents who reported more favorable views toward tobacco use enjoyment had markedly higher odds of cigarette tobacco use compared with those who strongly disagreed. However, because this study was cross‐sectional, these perceptions and tobacco use behavior were measured at the same time, and the observed association should not be interpreted as evidence of causality or temporal direction. It is possible that adolescents who already smoke are more likely to report favorable attitudes toward tobacco use, rather than these attitudes necessarily preceding tobacco use behavior. Therefore, these findings should be interpreted as correlational and hypothesis‐generating. This finding is consistent with previous research showing that favorable attitudes toward tobacco use and perceived social benefits are important correlates of tobacco use among adolescents [25]. Furthermore, the observed association between positive tobacco use expectancies and tobacco use suggests that tobacco use‐related beliefs may be relevant for future prevention and counter‐marketing strategies; however, the causal direction of this relationship should be clarified in longitudinal research.

Our cross‑sectional design precludes causal inference; nevertheless, the large, nationally representative GYTS sample and robust multivariable analyses enhance the generalizability and internal validity of our findings. We were unable to assess emerging products (e‑cigarettes, heated tobacco) in depth, an important direction for future waves of the GYTS given their rising popularity among Russian youth.

The implications of this research extend to both policy and future research directions. Regarding policy, the observed relationship between spending power and adolescent tobacco use underscores the efficacy of tax and price measures, specifically, escalating tobacco excise taxes to diminish affordability for this demographic [26]. Furthermore, the prominence of positive tobacco use expectancies necessitates the implementation of counter‐marketing campaigns designed to dismantle prevailing misconceptions about the social and emotional advantages of tobacco use [27].

In educational settings, school‐based interventions are crucial, particularly in middle and early high school grades, where curricula incorporating social norms approaches, peer‐led activities, and refusal‐skills training can be effectively implemented.

The Russian Federation has been a Party to the WHO FCTC since 2008 and has adopted tobacco‐control legislation that prohibits sales to minors and restricts tobacco sales near educational institutions [28]. Our findings suggest that stronger enforcement of these youth‐access measures, especially around schools, may help reduce tobacco use initiation among adolescents aged 13–15 years.

Our finding that positive tobacco use expectancies were strongly associated with tobacco use is consistent with studies showing that favorable tobacco use attitudes and perceived social benefits are important predictors of adolescent tobacco use. Evidence from other countries also indicates that peer influence plays a central role in shaping tobacco use‐related beliefs and behaviors, whereas school‐based interventions that include behavior change communication, peer components, and refusal‐skills training can strengthen anti‐tobacco use attitudes and reduce tobacco use among adolescents [29, 30].

Future research should adopt longitudinal designs to meticulously trace initiation trajectories, thereby providing a more nuanced understanding of the developmental processes involved. Additionally, the scope of inquiry must expand to include measures of emerging tobacco products, such as e‐cigarettes and heated tobacco, to comprehensively assess youth tobacco behaviors. Finally, the evaluation of recent policy alterations, including plain packaging and flavor bans, is paramount to determine their impact on youth tobacco use patterns.

## 5 Conclusion

Our study suggests that tobacco use among adolescents aged 13–15 years in the Russian Federation remains a significant public health challenge, characterized by rising prevalence with age, narrowing gender gaps, and strong socioeconomic and attitudinal predictors. Aligning with international and Russian‐specific literature, these findings underscore the urgency of comprehensive, multilevel tobacco‐control strategies tailored to the unique social and economic landscape of Russian youth.

## Author Contributions

**Ashfaq Ahmad Shah Bukhari** and **Syed Sikandar Shah** conceptualized and designed the study. **Ashfaq Ahmad Shah Bukhari** and **Syed Sikandar Shah** were responsible for data acquisition and supervision. **Mouhammed Sleiay** contributed to data analysis and interpretation. **Mahmoud M. Ali** drafted the manuscript, led the study, and performed the critical revision for important intellectual content. All authors reviewed, edited, and approved the final version of the manuscript.

## Ethics Statement

The researchers secured approval and licenses to access and reanalyze the GYTS data, in accordance with the ethical principles of the Helsinki Declaration (1964, 2008).

## Consent

Not applicable as we obtained the data from a publicly accessible database (https://extranet.who.int/ncdsmicrodata/index.php/catalog/958).

## Conflicts of Interest

The authors declare no conflicts of interest.

## Data Availability

Data are publicly accessible on an accessible database (https://extranet.who.int/ncdsmicrodata/index.php/catalog/958).
